# Effects of ten years organic and conventional farming on early seedling traits of evolving winter wheat composite cross populations

**DOI:** 10.1038/s41598-019-45300-1

**Published:** 2019-06-21

**Authors:** Vijaya Bhaskar A.V., Jörg Peter Baresel, Odette Weedon, Maria R. Finckh

**Affiliations:** 1 Crops, Environment & Land Use Programme, Crops Research Centre Oak Park, Teagasc, Carlow, R93 XE12 Ireland; 2 University of Kassel, Faculty of Organic Agricultural Sciences, Department of Ecological Plant Protection, Nordbahnhofstr. 1a, Witzenhausen, D-37213 Germany; 3 Technical University Munich, Institute for Plant Nutrition, Center of Life and Food Sciences Weihenstephan, Freising, 85354 Germany

**Keywords:** Agroecology, Agroecology, Plant breeding, Plant breeding

## Abstract

Early vigour traits of wheat composite cross populations (CCPs) based on high yielding (Y) or high quality (Q) or Y*Q varietal intercross evolving under organic or conventional conditions in parallel populations were studied hydroponically. To eliminate storage and year effects, frozen F_6_, F_10_, F_11_ and F_15_ seeds were multiplied in one field, resulting in the respective F_x.1_ generations. This eliminated generation and growing system effects on seed size for the F_6.1_ F_10.1_ and F_15.1._ Due to a severe winter kill affecting the F_11_, the generation effect persisted, leading to larger seeds and markedly different seedling traits in the F_11.1_ compared to the F_10.1_ and F_15.1_. Seedling traits were similar among parallel populations. Shoot length and weight increased in both systems until the F_11.1_ across farming systems and remained constant thereafter. Over time_,_ seminal root length and root weight of organic CCPs increased and total- and specific- root length decreased significantly compared to the conventional CCPs. Rooting patterns under organic conditions suggests better ability to reach deeper soil nutrients. In both systems, Q and YQ CCPs were more vigorous than Y CCPs, confirming genetic differences among populations. Overall, heterogeneous populations appear very plastic and selection pressure was stronger in organic systems.

## Introduction

Agriculture is facing the challenges of climate change, leading to higher variability and frequency of climatic extremes and increasing combined abiotic and biotic stresses^1,2^. To cope with these stresses, current farming systems are likely to require even more energy in the future (e.g. for irrigation systems and increased inputs) while facing reduced soil, water and air quality and overall system productivity^3^. An alternative is to develop agricultural systems with an inherent capacity to adapt to environmental changes and that contribute to the protection of soil organic content and structure^3,4^. This requires a major rethinking of breeding methods and redesigning of farming systems. In this context, evolutionary breeding using composite crosses has been gaining attention for direct selection in a specifically targeted environment^5–8^.

Composite cross populations (CCPs) are created by crossing a number of pure line varieties in all possible combinations. The progenies of the numerous crosses are then propagated as bulk populations under natural selection within specific farming environments^9–11^. Well-designed CCPs offer greater resilience to stressful and variable environments and provide for dynamic conservation of gene pools^10,12^. The higher intra-crop diversity found in CCPs allows for greater capability for evolution and adaptation to local conditions and farming systems including mixed cropping^5,11^, and may provide for a better response to continuously changing climatic conditions, pests and diseases^10,12–14^. As such, increased within-field variation and within-crop diversity can reduce the effects of larger environmental variability, on the one hand^15^. On the other hand, CCPs could effectively contribute to soil improvement via diverse organic residues and rhizodeposits. Thus, plant genotypes play a role “as a determinant of the species and genetic composition of the saprophytic microbial community resident to the rhizosphere”^16^. Specifically, wheat genotypes vary in their effects on microbial community composition^17^. Thus, CCPs should help increase microbial community diversity in the rhizosphere and by this farming system sustainability.

For successful organic cereal production, early vigour is of major importance to improve nutrient-use efficiency and weed competitive ability. Vigour may simply be described as rapid root and shoot growth^18^. Seminal roots developing directly from the germinating seed are responsible for initial absorption of water and nutrients^19^. They remain functionally active throughout crop growth^20^ and are also involved in the plastic response to growing environments^21^. Additionally, fast shoot growth increases crop competitiveness to weeds^22^. Exploring the strategies of plant seminal root system modification to acquire soil resources potentially allows for estimation of generative plant growth and yielding ability^21^, which in turn can assist in developing varieties with desired root traits for a particular environment^23^. Simulation studies have shown that deeper root systems are more likely to provide yield benefits in conditions where nutrients and water are scarce and heterogeneously-distributed within the soil matrix^24,25^. Assessing root-related features on a large number of plants over various genotypes *in-situ* is, however, tedious and technically challenging. As an alternative, artificial techniques like hydroponics provide a sensitive way to study early root characteristics under controlled conditions, and the results can be correlated with root development *in-situ*^19,26–28^.

In 2001, three winter wheat CCPs were created at the John Innes Centre (Norwich, UK) in co-operation with the Elm-Farm Research Centre (Newbury, UK)^29^. In autumn 2005, F_4_ seeds of all three CCPs were received by the University of Kassel, Germany. At the University of Kassel, each CCP was maintained under differing organic and conventional farming systems in 2005/06 (F_5_). Since the F_6_ (2006/07), each CCP was split into two parallel populations (I and II) within each farming system to determine the changes of the CCPs within the system and between systems. Populations were maintained in separated plots of >150 m^2^ in order to maintain an effective population size (N_e_) large enough to ensure genetic conservation^30^. Morphological and agronomic studies with these CCPs (F_5_ - F_15_) revealed that genetic variability is still high in the CCPs, even after 11 generations^31^ and unpublished molecular analyses by our laboratory confirm continuous recombination events in the populations. This allows for changes in their genetic make-up over time through selection and recombination and enables adaptation to their growing conditions^31^. The CCPs (F_11_) showed yield resilience in the face of a severe bare frost event in February 2012 that killed most parental varieties^32^. Yield stability of the organically grown CCPs was higher than for genetically uniform varieties under organic conditions, but not necessarily under conventional conditions^31^. In addition, in conditions of water-stress or drought the CCPs (F_14_, 2014/15) have out-yielded modern cultivars^31^.

The first indication of divergent evolution between organic and conventional CCPs for early vigour traits was shown by Bertholdsson *et al*.^33^ comparing seedlings of the F_6_ and F_11_ in a hydroponic system. The authors found differences in vigour traits due to the differing genetic composition of the CCPs, and due to organic and conventional farming systems. However, they used seeds that had been stored frozen for various lengths of time. The F_6_ was harvested in 2007 and had been stored frozen at −20 °C for 6 years, while the F_11_ (harvested 2012) was relatively fresh at the time of their experiment. In addition, there was a large difference in growing conditions, because the organic and conventional CCPs had been maintained in separate fields with variable soil quality. Furthermore, the effects of the winter kill 2012 were very strong on the CCPs of the F_11,_ and we later found that the legacy effect was carried over to the next generation (F_12_)^31^. Preliminary molecular analyses also confirmed changes in allele composition in the CCPs in the F_11_ as a result of the winter kill (unpublished).

By the F_15_, seeds stored frozen for 0 up to 10 years and grown under yearly changing weather conditions were available. Long-term seed storage influences seed vitality and, together with environmental differences during plant growth, may have implications for early vigour^34–37^.

To eliminate the effects of storage time, soil, and environmental conditions, seeds of the organic and conventional CCPs of the F_6_, F_10_, (before winter kill) F_11_ (after winter kill) and F_15_ were multiplied for one year in a common field under the same management system, and early vigour traits of the ensuing fresh and stored seeds were compared using the same hydroponic system as used by Bertholdsson *et al*.^33^. with the F_6_ and F_11_ before. This study comprises both methodical and fundamental aspects. In the first, methodical part, the following questions were addressed: (a) To which extent may the result of comparative analysis based on stored seeds harvested in different years and environments be biased by differences in seed size, germinability and early seedling vigour? (b) Does one year seed increase under identical conditions eliminate the system and year effects, and to which extent? In the second part, the effects of parental genetics and farming system over time on the early vigour of the wheat CCPs were assessed. The following hypotheses were tested: (a) Early vigour traits of CCPs change over time due to selection pressure by environmental factors; (b) Populations with different genetic background respond differently to selection pressure; (c) The extent and direction of the changes depends on the environmental conditions; we expect stronger selection pressure and an adaptation to low-input conditions in the populations maintained under organic management; (d) Extraordinary events such as natural severe frost may have strong effects on the genetic composition of a CCP, but in large enough populations there will remain enough genetic diversity to recover and to return close to the previous state.

## Results

### Comparative analysis of stored and fresh seeds for seed size

Storage and growing system interacted in their effects on the seed size (TGW) of the CCPs in the F_6,_ F_11_ and F_15._ For the F_10_, only storage had a significant effect on TGW (Table 1).

**Table 1:** Mean squares (MS) from analysis of variance of seed size comparing F_6_, F_10_, F_11_ and F_15_ grown in 2007, 2011, 2012, and 2016 in organic and conventional fields and stored frozen for 10, 6, 5, and 1 years, respectively, with fresh seed of the same generations (F_6.1_, F_10.1_, F_11.1_ and F_15.1_) increased for one year in the same field in 2017.

	Df	F_6_ vs F_6.1_	F_10_ vs F_10.1_	F_11_ vs F_11.1_	F_15_ vs F_15.1_
		MS	MS	MS	MS
Storage (T)	1	19.0^**^	780.0^***^	0.04	3.3
System (S)	1	44.0^***^	3.1	70.8^***^	24.5^**^
Population (P)	2	2.3	4.6	7.3	7.9^*^
T x S	1	22.2^**^	0.1	34.3^**^	23.1^**^
T x P	2	0.6	4.9	2.2	0.6
S x P	2	0.3	0.7	0.5	4.8
T x S x P	2	4.9	0.7	2.3	0.2

^*^P < 0.05, ^**^P < 0.01, ^***^P < 0.001.

The highest TGW (53.9 g) was recorded in the F_10_. In addition, in the F_6,_ F_11_ and F_15_, TGWs were significantly higher in the organic than in conventional CCPs (Table 2). The population effect on the seed size in the F_15_ and in the F_15.1_ (Table 1) was small and due to a difference between the Y CCPs (42.0 g) and the Q and YQ CCPs (both 43.3 g).

**Table 2:** Seed weights (TGW) of CCPs of F_6,_ F_10,_ F_11_ and F_15_ grown in 2007, 2011, 2012, and 2016 in organic and conventional fields, respectively and F_6.1_, F_10.1_, F_11.1_, and F_15.1_ grown in 2017 in a common conventional field.

	F_6_	F_6.1_	F_10_	F_10.1_	F_11_	F_11.1_	F_15_	F_15.1_
Organic CCPs	42.9a	42.7a	54.3	42.8	47.0a	44.7b	45.1a	42.4b
Conventional CCPs	38.3b	42.0a	53.5	42.2	41.2c	43.6b	41.1b	42.4b
Mean stored seeds	40.6 C		53.9 A		44.1B		43.1B	

Different lower case letters within a column indicate significant differences between organically and conventionally grown CCPs. Different upper case letters within a row indicate significant differences among generations grown in different years (stored seeds (P < 0.05).

### Effects of storage on seed germination and early vigour

Germination rates exceeded 95% and there were no significant differences (P > 0.05) among farming systems and CCPs or due to storage (Supplementary Table S1). The analysis of covariance of stored and fresh seeds showed that TGWs had very weak associations or did not significantly affect the seedling traits (P > 0.05) (Supplementary Table S2), hence seedling mean values were not adjusted according to TGW-based regression analyses.

Storage strongly affected seedling performance, and depending on generation, storage time interacted with the growing system or the populations or both (Table 3). Storage for 10 years (F_6_ versus F_6.1_) significantly reduced seminal root length (SRL) from 15.9 to 14.7 cm, total root length (TRL) from 78 to 62 cm and shoot dry weight (SDW) from 21.1 to 19.5 mg, respectively. Storage for six years (F_10_ versus F_10.1_) significantly interacted with growing system. It significantly reduced SRL and shoot length (SL) of the organic CCPs from 16.3 to 15.7 cm and 19.9 to 18.9 cm, respectively but not for the conventional CCPs. In contrast, root dry weight (RDW) and SDW of the conventional CCPs were significantly increased from 5.5 to 6.4 mg and 21.3 to 23.2 mg, respectively while no differences were observed for these parameters for the organic CCPs. Population effects also interacted with storage time where in most cases, the length and weight traits were significantly lower in the Y populations than in Q and YQ populations.

**Table 3:** Mean squares (MS) from analysis of variance of early vigour traits of seeds of the CCPs of the F_6,_ F_10,_ and F_11_ grown in 2007, 2011, 2012 in organic and conventional fields and stored frozen for 10, 6, and 5 years, respectively and the F_6.1_, F_10.1_, and F_11.1_ grown in 2017 in a common conventional field and not frozen before use. Relative values were analysed.

	DF	F_6_ versus F_6.1_ (10 years frozen)					F_10_ and F_10.1_ (6 years frozen)					F_11_ and F_11.1_ (5 years frozen)				
		SRL	TRL	SL	RDW	SDW	SRL	TRL	SL	RDW	SDW	SRL	TRL	SL	RDW	SDW
Storage (T)	1	^**^	^**^	NS	NS	^*^	NS	NS	NS	NS	NS	NS	^**^	NS	NS	NS
System (S)	1	NS	^**^	NS	^*^	NS	NS	^*^	NS	^***^	NS	^**^	NS	NS	NS	^*^
Population (P)	2	^***^	NS	^***^	^**^	^***^	^***^	NS	^***^	^***^	^***^	^**^	NS	^***^	NS	^***^
T x S	1	NS	NS	NS	NS	^*^	^***^	NS	^***^	^**^	^**^	NS	^*^	NS	NS	NS
T x P	2	NS	NS	NS	NS	NS	^*^	NS	^**^	^*^	NS	^**^	NS	NS	NS	NS
S x P	2	^*^	^*^	NS	^*^	NS	^**^	NS	NS	^*^	NS	NS	^*^	NS	NS	^*^
T x S x P	2	NS	NS	NS	^*^	NS	NS	NS	NS	NS	NS	NS	NS	NS	NS	NS

^*^P < 0.05, ^**^P < 0.01, ^***^P < 0.001, NS non-significant.

SRL - seminal root length; TRL – total root length; SL – shoot length; RDW– root dry weight; and SDW –shoot dry weight.

Storage of the F_11_ for 5 years again reduced TRL for both organic and conventional CCPs from 75 to 65 cm and from 77 to 63 cm, respectively. Storage time interacted with the populations only for SRL in a highly variable way. While in the F_11.1_ the Y populations (14.5 cm) had the shortest seminal roots compared to Q (15.5 cm) and YQ (15.4 cm), SRL of the Y populations (15.2 cm) in the F_11,_ was in between that of the YQ (14.9 cm) and Q populations (15.5 cm).

Overall, storage time clearly affected the outcome of the tests, therefore, to determine the effects of growing system and generation only comparisons among the freshly grown seeds are reported here (see Supplementary Table S3 for detailed comparisons among stored and fresh seeds).

Effects of generation, parental genetics, differing farming systems and their interactions on seed size and early vigour traits of fresh seeds.

**Figure 1:**
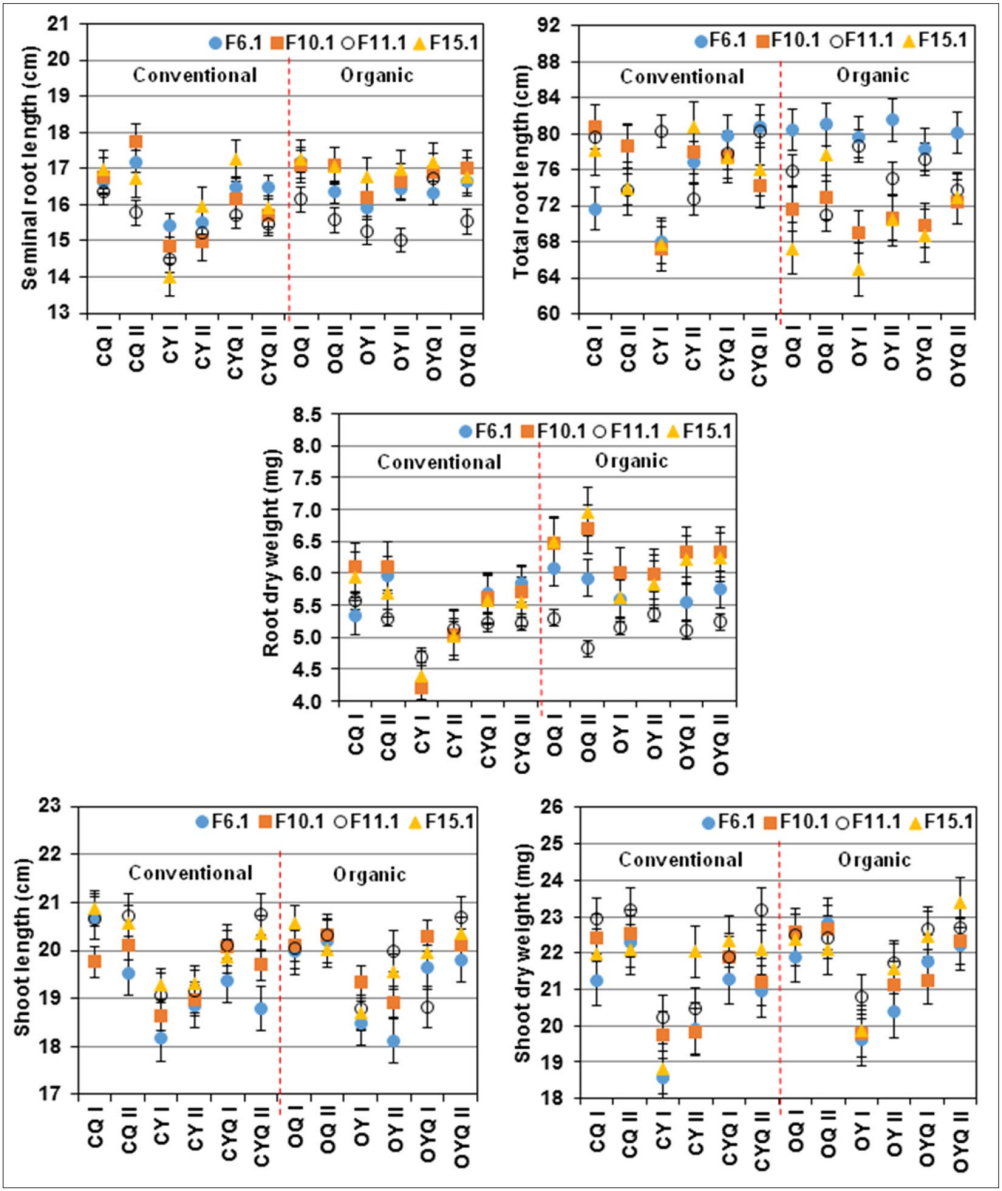
Seedling vigour traits from seeds of CCPs of four generation maintained in organic and conventional farming systems (absolute means of 8 replications).

The two parallel CCPs of Y, Q and YQ within generation and system were usually similar for all the length or weight traits (Fig. 1). An exception are the conventional Y populations. In every generation, the mean values for the length and weight traits were lower in the CY I than in CY II (Fig. 1). Compared to the frost affected F_11.1_, the mean values of root traits (SRL and RDW) of most CCPs in the F_10.1_ (before frost event) and in F_15.1_ (after frost event) were higher. In contrast, the shoot traits (SL and SDW) in the F_11.1_ were either similar or sometimes higher than F_10.1_ and F_15.1_ (Fig. 1).

Generation and system and, in most cases, system and population interacted for root traits. In contrast, only generation and population affected TGW and SL while SDW was additionally affected by growing system, with no interactions for these traits (Table 4).

**Table 4:** Mean squares (MS) from analysis of variance of seed size (absolute values) and early vigour traits (relative values) from seeds of CCPs of four generation (F_6.1_ to F_15.1_) grown under organic and conventional farming systems.

	Df	TGW	SRL	TRL	SL	RDW	SDW	SpecificRL	RDW:SDW
Generation (G)	3	^**^	^***^	NS	^***^	^*^	^***^	NS	^**^
System (S)	1	NS	^***^	^**^	NS	^***^	^*^	^***^	^***^
Population (P)	2	^*^	^***^	NS	^***^	^***^	^***^	^***^	NS
G x S	3	NS	^*^	^***^	NS	^***^	NS	^***^	^**^
G x P	6	NS	NS	NS	NS	^*^	NS	NS	^*^
S x P	2	NS	^***^	NS	NS	^***^	NS	^**^	^**^
G x S x P	6	NS	NS	NS	NS	NS	NS	NS	NS

^*^P < 0.05, ^**^P < 0.01, ^***^P < 0.001, NS non-significant.

TGW – thousand kernel weights SRL - seminal root length; TRL – total root length; SL – shoot length; RDW– root dry weight; SDW –shoot dry weight; SpecificRL - specific root length calculated as TRL/RDW; and RDW:SDW - root:shoot ratio.

**Figure 2:**
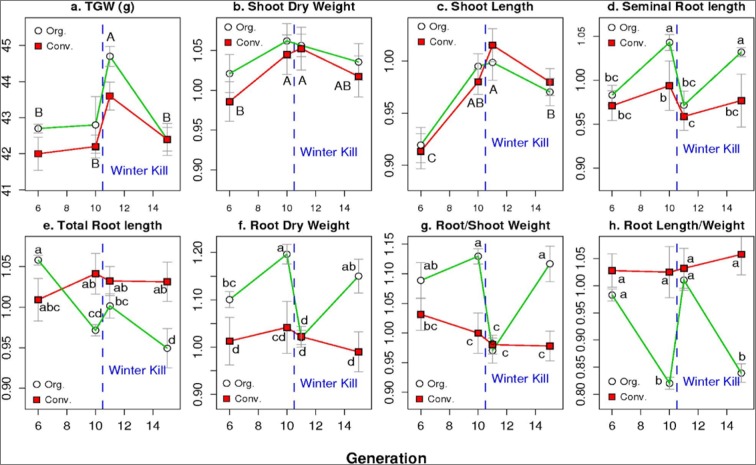
Mean values for seed size (TGW) and mean relative values for seedling traits as affected by the interactions of four generations (6.1, 10.1, 11.1, 15.1) and two farming systems, organic (Org.) or conventional (Conv.). Points within a panel marked with different lower case letters are significantly different from each other (P < 0.05). Where no Generation x system interactions occurred, different upper case letter within a panel refer to the mean values for each generation (i.e. the main effect).

**Figure 3:**
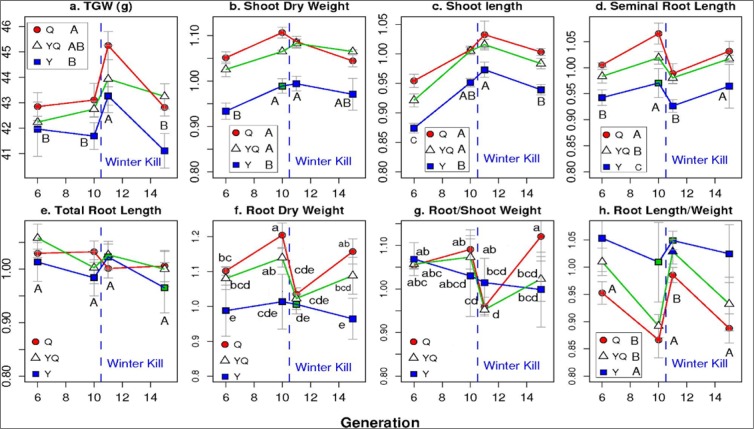
Mean values for seed size (TGW) and mean relative values for seedling traits as affected by the interactions of four generation (6.1, 10.1, 11.1, 15.1) and three populations (Y, Y = Yield, Q = Quality and YQ = Yield by Quality intercross). Points within a panel marked with different lower case letters are significantly different from each other (P < 0.05). Where no Generation x Population interactions occurred, different upper case letter within a panel refer to the mean values for each generation. Population main effects are indicated next to the legend.

Differences in TGW among farming systems were reduced to absent after growing for one year in the same field under identical conditions. However, while generation effects were no more evident for the F_6.1_ F_10.1_ and F_15.1_, TGW of the F_11.1_ was significantly greater than for the other three generations indicating a specific generation effect carrying through one year of seed increase (Fig. 2a). Among populations, the seed sizes in the Y populations were significantly lower than in Q, while YQ populations were intermediate (Fig. 3a**)**.

The SRL in the F_6.1_ were similar for the two systems (Fig. 2d). In contrast, in the F_6.1_, the organic CCPs had significantly higher RDW than the conventional CCPs (Fig. 2f). Concentrating on the F_10.1_ and F_15.1_ only, the organic CCPs had significantly increased SRL, compared to the F_6.1_ and the conventional CCPs. In contrast, SRL for the F_11.1_ was dramatically shorter in both systems than in the F_10.1_ and F_15.1_ indicating a strong effect of the frost event on the SRL even after an additional generation for seed increase. Contrary to SRL, TRL decreased over time in the organic populations but not in the conventional ones (Fig. 2e). Overall, this resulted in higher RDW and consequently lower specific root length (SpecificRL, i.e. TRL divided by RDW) in the organic populations, compared to the conventional ones in the F_10.1_ and F_15.1_ (Fig. 2f,h).

In general, differences among organic and conventional populations were obvious by the F_10.1_. The winterkill caused larger changes in the organic than in the conventional populations as seen in the F_11.1_. The F_15.1_ was similar to the F_10.1_ (Fig. 2a-h). Apparently, after the frost, genotypes with lower SRL and RDW were favoured while selection was in general towards higher TRL and SpecificRL in the organically managed populations.

Population effects interacted with RDW only due to the differential effects of the frost on the CCPs. RDW remained constant and low in the F_11.1_ for the Y CCPs. It was usually higher in the Q and YQ CCPs but dropped in the F_11.1_ to the level of the Y CCPs (Fig. 3f).

**Figure 4:**
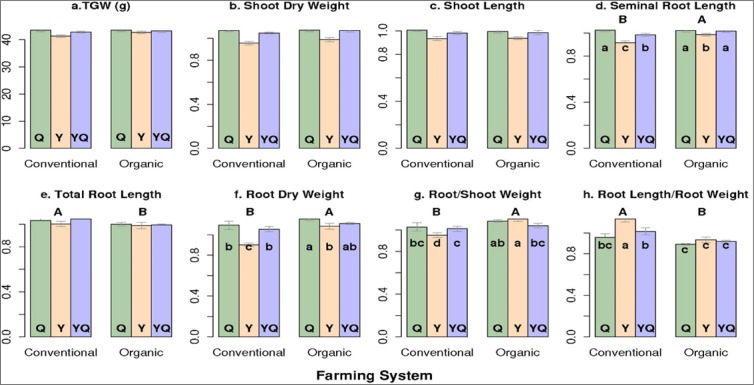
Mean values for seed size (TGW) and mean relative values for seedling traits as affected by the interactions of three populations (Y, Y = Yield, Q = Quality and YQ = Yield by Quality intercrossed) and two farming systems. Columns within a panel marked with different lower case letters are significantly different from each other (P < 0.05). System main effects are indicated with different upper case letters.

SRL did not differ for the organic and conventional Q CCPs but it was considerably shorter in the conventional Y CCPs than in the organic ones (Figs 1 and 4d). The organic YQ CCPs had SRL equal to the organic Q CCPs while in the conventional system SRL of the YQ CCPs was intermediate (Fig. 4d). As to be expected, the system specific effects on SRL carried through into the RDW and SpecificRL (Fig. 4f,h).

There was a tendency of SL and SDW to increase from the F_6.1_ to the F_11.1_ for both systems, remaining at these higher values thereafter (Figs 1 and 2b,c). They were consistently higher in the Q and YQ populations than in the Y populations (Figs 1 and 3b,c). The growing system did not affect SL and effects on SDW were small for both, the conventional and organic populations. The root to shoot ratios interacted following the interactions of the root traits with strong effects of the F_11.1_ and the conventional Y CCP (Figs 2g, 3g and 4g). Detailed corresponding absolute mean values for seedling traits are given in Supplementary Fig. S1.

## Discussion

This study was based on and extends previous research of Bertholdsson *et al*.^33^ addressing the effects of organic and conventional management on the early vigour traits across ten generations of evolving CCPs. In addition, by comparing fresh and stored seeds, the effects of various lengths of storage time on seedling performances could be analysed.

Firstly, the larger seed size in the organic CCPs in comparison to the conventional CCPs of the F_6_ to the F_11_ was the likely result of superior soil quality of the organic field. However, the seed size was still lower in the conventional than in the organic CCPs in the F_15_, despite that both had been grown in the same soil type and quality since the F_13_. The environmental differences causing variation in seed size among growing systems were removed in the fresh seeds of F_6.1_, F_10.1,_ F_11.1_ and F_15.1_ grown for one year in a common field under identical conditions. Similarly, seed size differences among a number of CCPs of F_13_ originating from different countries disappeared in the F_14_ when seeds were produced in the same field under the same management system^31^. This suggests that the organic farming system favoured the production of larger seeds. Seed size heritability is low^38^ and variation in seed size tends to be a result of phenotypic plasticity, which is thought to be adaptive, especially as the result of environmental variation^39,40^.

Seed multiplication under identical conditions, however, did not eliminate the year effects in the F_11.1,_ grown from the F_11_ that had been exposed to an exceptional frost event^32^. Thus, carryover effects of the frost event in 2011/12 persisted for at least one additional year. Nevertheless, the large genetic variability in the CCPs effectively overcame this one-off climatic stress, as there were no more signs of frost effects on the seed size nor on early seedling traits in the F_15.1_ (after frost event) and their trends were similar to that of F_10.1_ (before frost event)_._ The frost event seriously reduced the effective population size for one year^30^ and preliminary molecular analyses suggest that the changes due to frost disappeared within two years (unpublished). A longer time period with higher selection pressures may induce permanent changes on phenotypic traits, however^41^.

Storage time, especially above five years affected seedling root and shoot growth in a highly variable way confirming previous results^34,35^. Nevertheless, our results from fresh seeds were similar to the results of Bertholdsson *et al*.^33^. Clearly, it is not recommended to use wheat seeds stored for more than five years for studying important vigour traits.

In the fresh seeds of F_6.1_ to the F_15.1_, within system, the parallel but separate populations followed similar evolutionary trajectories, suggesting that the observed changes are more related to the effects of differing farming systems. However, CY I in particular, showed a different trajectory for all the measured seedling traits in every generation, compared to CY II, indicating that the CY I is more reactive to the growing systems than all other conventional CCPs and it seems less stable, compared to CY II. These results also suggest that the two conventional Y CCPs may have undertaken divergent evolution over time. This supports observations of divergent trajectories in the conventional Y CCPs by Weedon^31^ who compared the agronomic performance of all 12 CCPs across eleven generations (F_5_ - F_15_). In general, variations among populations and between generations are attributed mainly to environmental stress, plant-plant competition and plant-pathogen co-evolution^42^. The populations were all grown within less than 100 m from each other in each year and we have no explanation as to why the CY I and CY II populations diverge.

Root systems and their adjustments are considered as a key aspect of a plant’s phenotypic plasticity in response to its growing conditions^43^. Phenotypic plasticity, however, could itself be under genetic control and thus subjected to selective pressure^44^. Diverse populations like CCPs could respond to variable growing environments by becoming both more plastic and genetically variable^15^. The CCPs exposed to differing organic and conventional growing conditions were able to produce significantly higher root dry weight in the organic CCPs after only one generation of maintenance, demonstrating great plasticity and adaptability. Clear farming systems effects were observed both after four years (F_10.1_) and after nine years (F_15.1_), where seminal root length and root dry weight significantly increased in the organic CCPs in comparison to the conventional ones.

The other key feature of root adjustments between organic and conventional CCPs were the changes related to the total root length and specific root length. Total root length affects the distribution of roots in the soil profile^45^, whereas specific root length indicates root length for a given root mass, and is a key indicator of root thickness^46^. Generally, finest roots, with diameter 0 to 0.25 mm and thinner or finer roots, with diameter 0.25 to 0.5 mm account for a substantial portion of the total root length and total root surface area. For crops under conventional systems, early vigour may not be that important, and as such investment in shallow roots is relatively cheap as mineral nitrogen is easily accessible in the topsoil^47^. In our study, conventional CCPs in all the years had a high specific root length as a result of greater total root length and lower root dry weights. Root systems of this kind have a lower soil penetrating ability and a fast growth rate leading to quick loss of nutrients and incomplete exploration of deeper soil resources, as a result of adaptation to high rates of mineral nitrogen. In contrast, investment in expensive long-lived roots with slower metabolic rates is critical to organic farming^48^. This can be substantiated by our study, where the CCPs exposed to organic growing systems had very different rooting patterns over time. Longer seminal roots and a very low specific root length as a result of decreased total root length and higher root dry weights of the organic CCPs indicate longer, thicker, heavier and steeper main root systems. These traits may bring the crop root systems into greater contact with deeper soil layers with heterogeneous conditions of low nutrient supply, and where mineralization of organic materials is rather slow, due to low soil temperatures in spring^49,50^. These root features may also be optimal to invest in a relationship with mycorrhizal fungi for indirect nutrient uptake^51^. When comparing to either organically-bred or conventionally-bred varieties, we found that the three organic CCPs of the F_15.1_ clearly stood out with lower specific root lengths^52^. This suggests that adaptive processes within the organic CCPs have led to very specific changes that have not been achieved so far by breeding for organic farming systems using the conventional line breeding approach.

Root growth positively influences shoot growth^22^, and as such, fast shoot growth in conditions where no herbicides are allowed can improve canopy formation and biomass production by exploiting available solar radiation, which in turn exerts greater weed competitive ability^53^. In addition, net primary productivity is increased, further enhancing the translocation flow of assimilates to the roots^54,55^. These observed root traits on young plants may regulate the architecture of the plant systems at the adult crop stages, improving yielding ability in the organic systems. Greater seedling vigour in root and shoot growth enhances nitrogen uptake in wheat^56^, and rooting depth of winter wheat genotypes improves nitrogen uptake^49,57^. Overall, heterogeneous populations appear very plastic and show an apparent adaptation to local growing environments.

The differences on root vigour observed between OY and CY show that they respond very differently to the farming systems that they were grown in. From the F_6.1_ to the F_15.1_, both organic and conventional CCPs produced for yield had lower early vigour than the Q and YQ CCPs indicating genetic effects of the parents on the performance of the CCPs. This also highlights that there is genetic variation for nutrient-use efficiency (i.e. utilizing available nutrients from soil or fertilizers more effectively) within the CCPs confirming the results of Bertholdsson *et al*.^33^. The interaction with the growing system highlights that CCPs are capable of adapting to differing farming systems^31^. The tendency of the Q and YQ populations of the organic system towards greater vigour in root growth in comparison to the Q and YQ populations of the conventional system was further supported by field observations comparing the organic CCPs^52^. It is, however, difficult to decide whether the observed differences are transitory or long-lasting. Generally, weather conditions were the same for each population in each generation, but site-specific environmental conditions varied such as soil quality (specifically challenging for the conventional CCPs until F_12_), disease loads and nutrient availability that might have affected the populations over time.

Despite seed increase under the same conditions, the dramatic effects of the extreme frost event on the CCPs of the F_11_ were carried through to the fresh seeds of F_11.1._ Only in the F_11.1_, were there no system differences for root traits that were very prominent in the other three generations. An overriding effect of the frost event was a very low ratio of root to shoot weights or nutrient uptake rates per unit mass. Such conditions with proportionately higher shoot growth than root growth, especially at an early stage may result in poor anchorage and suboptimal acquisition of soil resources^43^, which in turn leads to inferior crop structure with a sparse canopy, limiting successional crop developmental stages^58,59^. It is, therefore, not so surprising that the frost affected changes in root traits seem magnified in the organic CCPs, in which SRL was increased by the F_10.1_. In contrast, the conventional CCPs apparently invested more in above than below ground biomass with little changes in root traits. Nevertheless, this one-off climatic event did not change the entire genetic makeup of the populations, as evidenced by the again improved early root vigour in the organic F_15.1_ (four years after the frost event) with trends were similar to the F_10.1_ (before the frost event)_._

## Conclusions

This study highlights that wheat seeds stored for >5 years appear unsuitable for directly studying important seedling traits. While differences in seed size can usually be eliminated by the same management in a single year, this was not true for the effects of an extreme frost event. Nevertheless, the marked effect of the winterkill was short term and did not persist.

Most seedling traits were similar among the parallel populations, suggesting that evolution was driven by the initial genetic composition and farming system. The only exception were the two conventional Y populations that apparently have experienced separate random events in an early generation^31^. Selection pressure was overall stronger in organic farming systems. The evolutionary changes of organic CCPs over time resulted mainly in longer, thicker and a deeper main root systems that may be better adapted to conditions of limited nutrient supply that usually prevail from fall to late spring under organic management. In contrast, under conventional management, very little or no changes were evident. The differences between CCPs created from the Y or Q parents or the YQ intercross demonstrate the clear genetic effects of the parents on the performance of the CCPs Overall, the ability of CCPs to adapt their seedling characteristics, particularly when grown under an organic system, and their ability to buffer a single extreme climatic event demonstrate the high degree of resilience of the CCPs. Thus, CCPs as such, provide a useful breeding base for specifically targeted environments for improved agricultural production. In addition, however, when used as populations, resilience may be further enhanced by the fact that there is a moderate degree of heterosis within the CCPs that may lead to superior performance of newly crossed individuals enhancing the CCP performance. Hydroponics offers a cost efficient, quick, and thorough tool for root measurements that highly correlate with the results of field experiments, making it a valuable selection tool for breeders.

## Materials and Methods

### Plant material

The wheat composite cross populations (CCPs) were created in 2001 by The Organic Research Centre, Elm farm and the John Innes Institute in the UK through a complete half-diallel cross of twenty wheat varieties. Varietal release dates ranged from 1934–2000 and varieties were chosen based on their agronomic performance under organic or low-input conditions in Europe and the UK^29^, as well as to ensure a broad genetic base for the CCPs^60,61^. The progeny of the crossings was multiplied for one season in a greenhouse before being bulked into three separate CCPs. The first CCP (Q, Q = Quality) was created by bulking the F_2_ of 12 high baking quality parents. The second CCP (Y, Y = Yield) was based on the crosses of 9 high yielding parents. The parental variety ‘Bezostaya’ is known for both high yielding and high baking quality and was included in both Y and Q CCPs. The third CCP (YQ, YQ = Yield x Quality) is a product of crossing 8 Y x 11 Q parents, plus all these 19 parents crossed with Bezostaya^32^. One of a total of four male sterile lines was crossed with each parent in addition to the half-diallel crosses, and aliquots of their progenies bulked into the three CCPs according to the parents used to encourage outcrossing within the CCPs^61^. The CCPs were multiplied at four sites across the UK until the F_4_. In 2005, seed of the F_4_ from each CCP was bulked across sites and from this a portion was sent to the University of Kassel. Since 2005 (F_5_), the three populations (Y, Q and YQ) have been maintained there under organic (O) and conventional (C) growing conditions without conscious selection, apart from removal of plants taller than 130 cm in the first few years. In 2006/07 (F_6_), each CCP was split into two parts (I and II) within farming system, resulting in a total of 6 organic CCPs and 6 conventional CCPs that were managed separately in parallel in separate plots of at least 150 m^2^ to avoid genetic drift. Every year, 1 kg seeds of each CCP was stored frozen at −20 °C for future analyses. These parallel populations are separate and distinct allowing for the comparison of evolutionary changes over time within and between organic or conventional farming systems^31–33^.

### Field conditions

The winter wheat CCPs were maintained at the University of Kassel research fields in Neu Eichenberg situated at 51°22″N latitude, 09°54″E longitude at 247 m above sea level, since 2005. Mean annual temperature during these years was 9.6 °C (Range: 8.4–11.2 °C) and mean annual precipitation 679 mm (Range: 540–791 mm)^31^. A particularly extreme climatic event occurred in winter 2011/12 during the F_11_ when after a frost-free winter until the end of January, temperatures suddenly dropped within one night by more than 20 °C followed by two weeks of bare frost with night temperatures below −15 °C. This resulted in soil frozen to a depth of 50 cm. This period was followed by extremely dry and warm weather from mid-February to March 2012 exposing plants in the frozen soil to severe water-stress. The CCPs were damaged but recovered well in contrast to most of the parental varieties that suffered from winterkill^32^.

The certified organic field maintained since 1984, is a fine loamy loess soil (deep Haplic Luvisol) with about 80 soil points according to the German soil grading scale (0–100) where the points roughly indicate the innate soil fertility with respect to potential wheat yield under optimal conditions and 1 point equals about 100 kg. The CCPs were usually grown after building soil-fertility with grass/clover ley for two years. No artificial fertilizers nor pesticides were applied. Mechanical weed control was performed before winter and/or at tillering. Until the F_12_, the conventional field was a sandy loam loess soil (Stagnic Luvisol) with 60 soil points at about 500 m distance from the organic field. Yields of wheat in the conventional field were either similar or lower than yields achieved on the organic field, despite the use of mineral fertilizer input highlighting the lower yield potential of the site^31^. The conventional populations were rotated yearly with grass/clover. No fungicides or insecticides were applied, but herbicides were used once a year in early spring. Mineral fertilizer was applied in two split doses at the rate of 50 kg N/ha and 75 kg N/ha during the growing season. Growth regulators were used only in 2008 and 2009^32^. Since the F_13_ (2013/14), the conventional populations have been grown in a conventionally managed field adjacent to the organic field with the same soil type as the organic field.

In 2016/17, stored seeds of the 12 CCPs of the F_6_, F_10_, F_11_ and F_15_ were propagated in a common field at the Dürnast research station of the Technical University of Munich, Freising (11°41′60″E, 48°23′60″N), resulting in fresh seeds of F_6.1_, F_10.1_, F_11.1_ and F_15.1_ that were used in this study. The mean temperature and precipitation during the growing season (September 2016 to August 2017) were 9.0 °C and 749 mm, respectively. The soil is characterized as mostly homogeneous silty clay loam (Cambisol) with about 66 soil points, and a water holding capacity of about 160 mm within a soil depth of 1 m. The pre-crop was a grass/clover mixture. The CCPs were grown in 6 m^2^ plots and received 180 kg N/ha mineral fertilizers applied as two split application during the growing season. In addition, fungicide (Input®Xpro + Skyway®Xpro or Ceriax®), herbicide (Roundup®PowerFlex or Artus®), insecticide (Karate Zeon) and growth regulators (Cycocel Chlormequat Chloride (CCC) or Moddus®) were applied.

### Hydroponic system

The early seedling traits were assessed in the autumn/winter 2017/2018 using a hydroponic system based on Bertholdsson *et al*.^33^. Seeds were grown in containers (20 L) filled with a balanced complete nutrient solution with a phosphate buffer (pH 6.5)^62^. Stock solutions of macronutrients (per 2 L: Na_2_HPO_4_.2H_2_O - 39 g, KH_2_PO_4_ - 68 g, KCL - 37 g, MgSO_4_.7H_2_O - 61 g, Ca(NO_3_)_2_ - 118 g) and micronutrients (per L: FeCl_3_.6H_2_0 - 27.53 g, MnCl_2_.4H_2_O - 1.39 g, ZnSO_4_.7H_2_O - 0.86 g, CuSO_4_.5H_2_O - 0.20 g; H_3_BO_3_ - 0.099 g and Na_2_MoO_4_.2H_2_0 - 3.40 g) were prepared individually. In the 20 L container, 200 ml of the macronutrient and 100 ml of the micronutrient stock solution were added and filled with deionized water (2 mM N concentration). The seeds were placed in strips of corrugated cardboard with the embryo facing down towards the solution and with 10 mm within row spacing. The lower part of the cardboard was ironed beforehand to prevent seeds from falling down. Filter paper strips were used as wicks and suspended over special frames into the hydroponic containers. The nutrient solution was renewed after seven and ten days and aerated by continuous bubbling of air through the solution. The plants were grown in a greenhouse with 18/12 °C (day/night) temperature regime for 14 days at a photoperiod of 16 h supplemented with artificial lighting to maintain a minimum light intensity of 250 μ mol quanta m^−2^ s^−1^.

Per container, 14 rows of 12 seeds each could be accommodated. The two outer rows were used for standard plants and always sown with two pure lines (the varieties ‘Achat’ and ‘Tobias’), and the edge seedlings per row were used as border plants. It was only possible to process a maximum of two containers per day. Thus, only 24 populations could be grown at the same time. The seeds of the F_15_ were not used for germination tests or seedling traits comparison in the hydroponic system, as the one-year storage should not have substantial effects on seedling traits. However, the seed size of F_15_ was assessed to understand the field and year effects, as from the F_13_, both organic and conventional CCPs have been maintained in same soil quality^31^. Thus with four generations, 12 CCPs per generation and stored and fresh seeds for the three older generations to be tested there were thus a total of 84 CCPs to compare.

An important question was to determine if the parallel CCPs had evolved similarly. The second most important question was to understand the growing systems effects. In order to allow for the detection of small differences among parallel CCPs and among growing systems, the 12 CCPs of a given generation were therefore grouped within box. To determine the differential effects of the different storage times involved as the second most sensitive difference expected, the stored and the frozen seeds of the CCPs were paired within date in order to clearly show the storage effects. Evolutionary effects over time should be the most robust if they are of relevance. Therefore the generation effects were tested across the whole testing period. This was achieved by normalizing all data against the standard plants (see below). The eight replicates per generation were grown within two days each aver periods of about four weeks that are referred to as four separate experiments.

The 12 CCPs of a given generation were randomized as rows of 10 seeds within the container and each generation was replicated eight times. Every two days one replicate consisting of two containers was sown to allow sufficient time for handling and processing the plant samples after each harvest.

### Measurements

Seed germination rates were assessed twice using 25 seeds per CCP in Petri-dishes with dense filter paper (LLG-Labware®) moistened with 2 ml deionized water. Petri-dishes were placed at 20 °C, for 8 days and germinating seeds counted after four and eight days. The 1000 kernel weights (TGW) of each CCP were determined based on three times 100 seeds.

At the end of the hydroponic experiment, the length of the longest seminal root (SRL) and the shoot length (SL) from the scutellar node to the tip of the highest leaf were measured on each plant directly using a ruler. Subsequently, the total root length (TRL) (sum of the lengths of all roots in the seminal root system) per plant was determined with an Epson photo scanner (Epson Perfection V700 with 6400 dpi resolution) (Epson, Long Beach, CA) and using WinRHIZO Pro image analysis system (Regent Instruments, Inc., Quebec City, QC). Dry weights of roots (RDW) and shoots (SDW) were determined after drying at 105°C overnight. Specific root length (SpecificRL) was calculated as TRL/RDW and root:shoot ratio calculated as RDW/SDW.

### Data analysis

As the experiments were distributed across two containers per replicate, and replicates were sown at different times, prior to the analyses, the measured seedling traits of the 12 CCPs in each container of every generation were divided by the standard, i.e. the mean of the varieties ‘Achat’ and ‘Tobias’ that were sown in every box, resulting in mean values relative to the standard. The two reference varieties behaved very similar over time across all experiments with remarkable little variation (Supplementary Fig. 2).

A linear mixed-effects model was developed using R, version 3.2.2^63^ and the lmer function of the lme4 package^64^. Seedling traits were assessed with generation, system and population as fixed effects and experiment and replicates as random effects. The model thus accounted for per-experiment random adjustment to the fixed intercept as well as per-replicate random adjustments to the slopes of predictors varying within the subject. Normality and equal variance of data were tested by using residual and graphing methods. When the interaction effect was significant (P < 0.05) posthoc Duncan tests were used to examine the differences. We report both relative and absolute mean values of the seedling traits.

## Supplementary information

Effects of ten years organic and conventional farming on early seedling traits of evolving winter wheat composite cross populations
