# Supplementary material for: Effects of ten years organic and conventional farming on early seedling traits of evolving winter wheat composite cross populations
Source: Sci Rep. 2019 Jun 21;9:9053. doi: 10.1038/s41598-019-45300-1 (PMC6588703; doi:10.1038/s41598-019-45300-1)
Supplement: Supplementary file 1 — Effects of ten years organic and conventional farming on early seedling traits of evolving winter wheat composite cross populations [file 41598_2019_45300_MOESM1_ESM.pdf]

Effects of ten years organic and conventional farming on early seedling traits of evolving winter wheat composite cross populations

Vijaya Bhaskar AV[1, 2*], Jörg Peter Baresel[3], Odette Weedon[2], Maria R Finckh[2]

[1]Crops, Environment & Land Use Programme, Crops Research Centre Oak Park, Teagasc, Carlow R93 XE12, Ireland

[2]University of Kassel, Faculty of Organic Agricultural Sciences, Department of Ecological Plant Protection, Nordbahnhofstr. 1a, Witzenhausen, D-37213, Germany

[3]Technical University Munich, Institute for Plant Nutrition, Center of Life and Food Sciences Weihenstephan, Freising, 85354, Germany

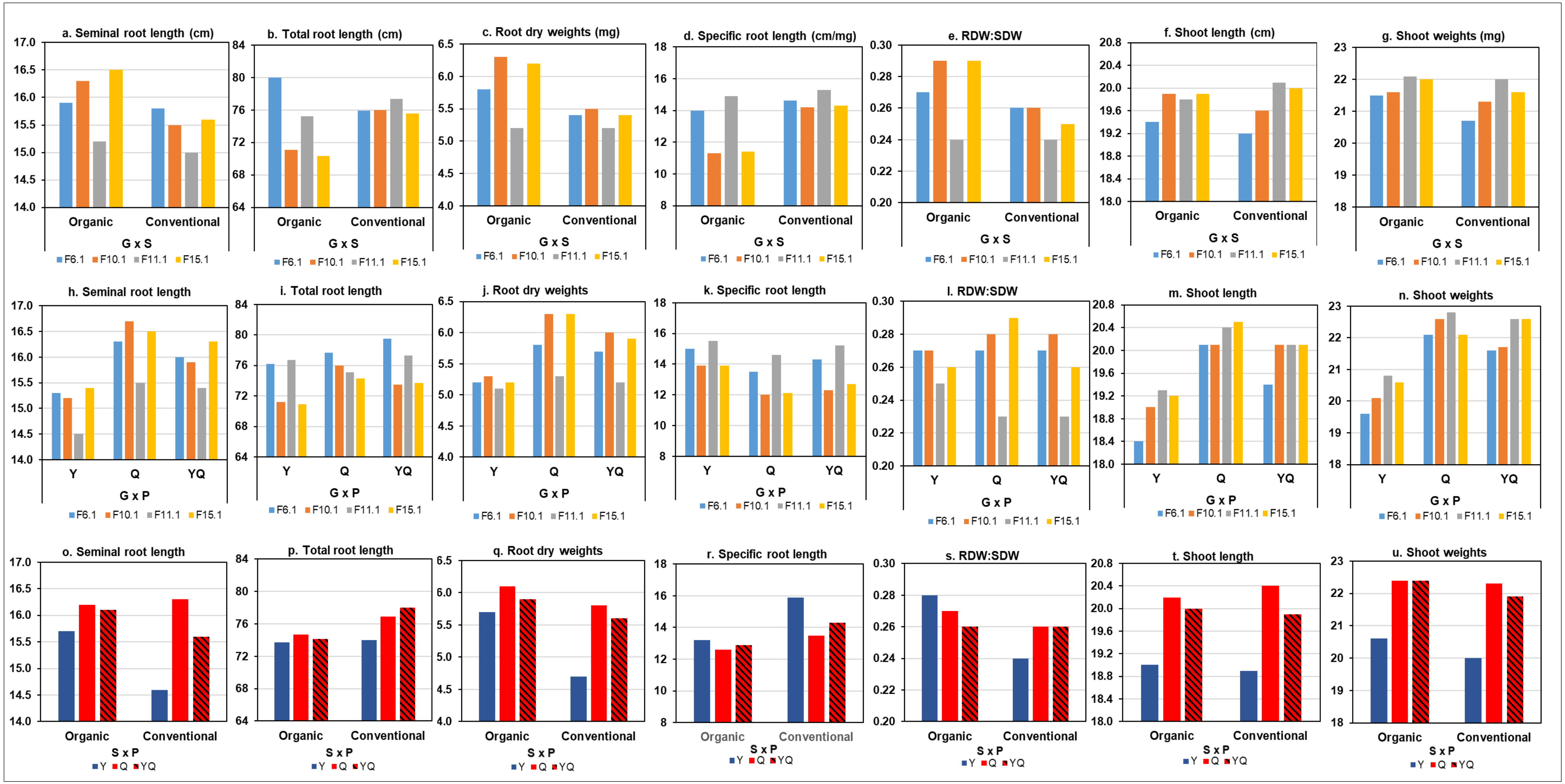

Figure S1: Absolute mean values for seedling traits of CCPs of the $F_{6.1}$, $F_{10.1}$, $F_{11.1}$, and $F_{15.1}$ as affected by the interactions of generation (G), system (S) and population (P). Y: yield CCPs, Q: quality CCPs and YQ: yield by quality intercrossed CCPs

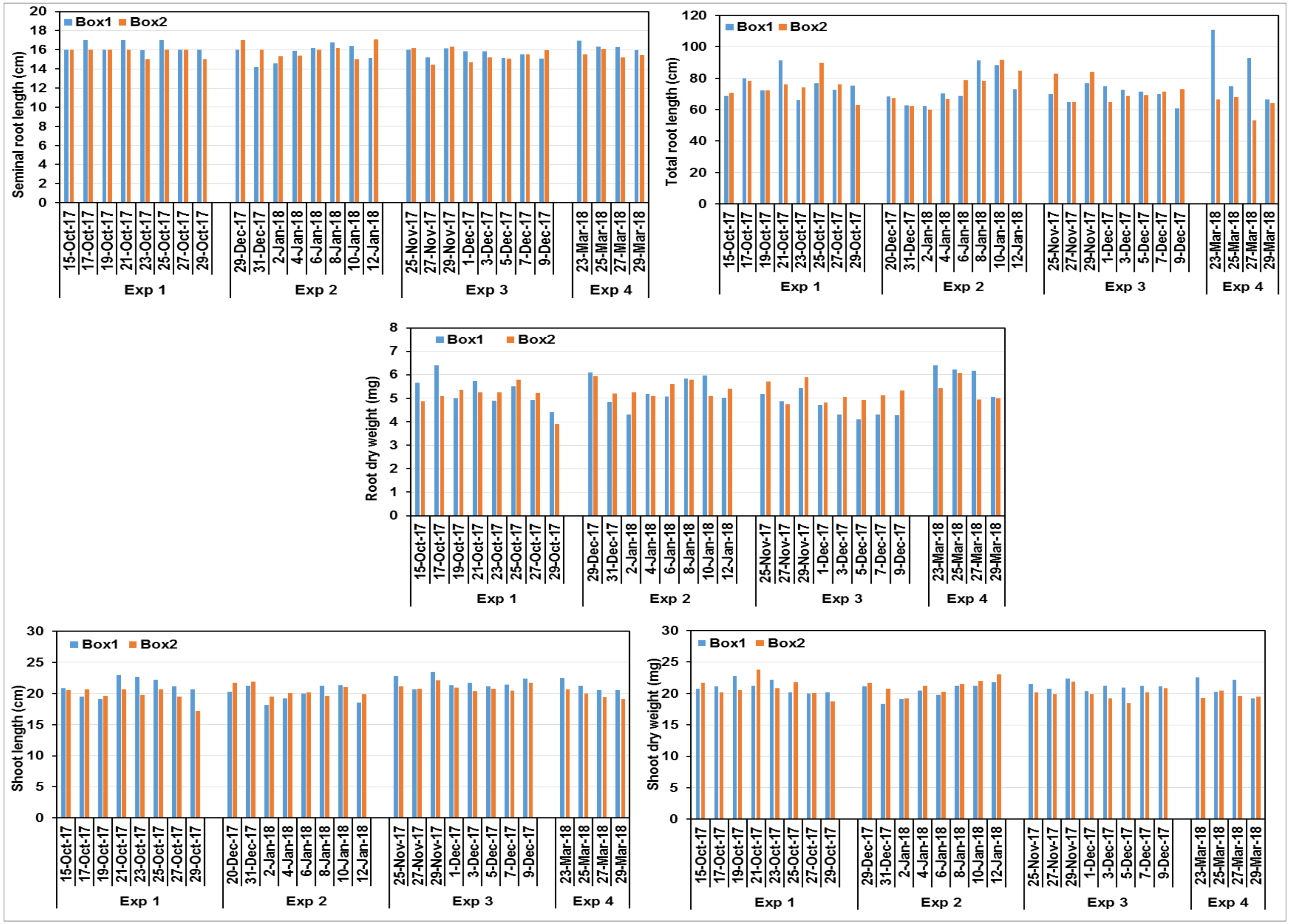

Figure S2: Mean values for seedling traits of reference varieties, Achat and Tobias per box. Experimental (Exp) periods are: F_6 and $F_{6.1}$= Exp 1, F_{11} and $F_{11.1}$= Exp 2, F_{10} and $F_{10.1}$ = Exp 3, and only $F_{15.1}$ = Exp 4

Table S1. Mean squares (MS) from analysis of variance of germination of seeds of CCPs of the $F_{6,}$ $F_{10,}$ and F_{11} grown in 2007, 2011, 2012 in organic and conventional fields and stored frozen for 10, 6, and 5 years, respectively and the $F_{6.1}$, $F_{10.1}$, $F_{11.1}$ and $F_{15.1}$ grown in 2017 in a common conventional field. In addition, the MS for the comparisons among the fresh seeds are given (right column).

	F_6 versus $F_{6.1}$ (10 years frozen)					F_{10} and $F_{10.1}$ (6 years frozen)				F_{11} and $F_{11.1}$ (5 years frozen)				$F_{6.1}$ $F_{10.1,}$ $F_{11.1}$ and $F_{15.1}$				
	Day 4			Day 8		Day 4		Day 8		Day 4		Day 8		Day 4			Day 8	
	DF	MS	P	MS	P	MS	P	MS	P	MS	P	MS	P	DF	MS	P	MS	P
Generation (G)	1	0.09	0.5	1.0	0.09	0.8	0.09	1.0	0.06	0.01	0.9	0.04	0.7	3	0.7	0.3	0.3	0.4
System (S)	1	0.8	0.09	1.0	0.09	0.09	0.5	0.0	1.0	0.09	0.7	0.4	0.3	1	0.3	0.4	0.02	0.8
Population (P)	2	0.3	0.3	0.07	0.8	0.2	0.4	0.4	0.3	0.3	0.7	0.2	0.5	2	0.4	0.4	0.1	0.7
G x S	1	0.3	0.3	0.2	0.5	0.09	0.5	0.2	0.4	0.8	0.3	0.04	0.7	3	0.04	1.0	0.3	0.4
G x P	2	0.1	0.6	0.4	0.3	0.03	0.9	0.07	0.8	0.8	0.4	0.07	0.8	6	0.4	0.5	0.1	0.8
S x P	2	0.1	0.6	0.07	0.8	0.09	0.7	0.09	0.7	0.2	0.7	0.09	0.7	2	0.1	0.8	0.1	0.7
G x S x P	2	0.04	0.8	0.4	0.3	0.03	0.9	0.07	0.8	0.7	0.4	0.07	0.8	6	0.3	0.7	0.09	0.9

Table S2. Analysis of covariance of seed size with seedling traits in the seeds of CCPs of $F_{6,}$ F_{10} and F_{11} grown in 2007, 2011 and 2012 in organic and conventional fields, respectively and $F_{6.1}$, $F_{10.1}$, $F_{11.1}$, and $F_{15.1}$ grown in 2017 in a common conventional field.

	SRL		TRL		RDW		SL		SDW		SpecificRL		RDW:SDW	
	r^2	P	r^2	P	r^2	P	r^2	P	r^2	P	r^2	P	r^2	P
F_6 and $F_{6.1}$	0.13	<0.001	0.14	<0.001	0.04	0.004	0.001	0.62	0.03	0.011				
F_{10} and $F_{10.1}$	0.002	0.54	0.19	0.06	0.14	<0.001	0.016	0.08	0.06	<0.001				
F_{11} and $F_{11.1}$	0.04	0.004	0.00001	0.96	0.0004	0.79	0.002	0.54	0.03	0.02				
$F_{6.1}$, $F_{10.1,}$ $F_{11.1}$ and $F_{15.1}$	0.015	0.02	0.002	0.44	0.005	0.19	0.009	0.06	0.05	<0.001	0.001	0.57	0.003	0.30

SRL - seminal root length; TRL – total root length; RDW– root dry weight; SL – shoot length; SDW –shoot dry weight; SpecificRL - specific root length calculated as TRL/RDW; and RDW:SDW - root:shoot ratio.

Table S3. Effects of organic and conventional farming systems on early seedling traits of the CCPs of the $F_{6,}$ $F_{10,}$ and F_{11} grown in 2007, 2011, 2012 in organic and conventional fields and stored frozen for 10, 6, and 5 years, respectively and the $F_{6.1}$, $F_{10.1}$, and $F_{11.1}$ grown in 2017 in a common conventional field. Relative and absolute (in parentheses) values are shown. Significant results are given in bold.

	SRL (cm)		TRL (cm)		SL (cm)		RDW (mg)		SDW (mg)	
	F_{6}	$F_{6.1}$	F_{6}	$F_{6.1}$	F_{6}	$F_{6.1}$	F_{6}	$F_{6.1}$	F_{6}	$F_{6.1}$
**G x S**										
Organic	0.95	0.98	0.90	1.06	0.88	0.92	1.15	1.10	**0.91b**	**1.02a**
	(14.7)	(16.0)	(63.3)	(80.2)	(19.3)	(19.4)	(5.3)	(5.8)	**(19.2)**	**(21.5)**
Conventional	0.95	0.97	0.86	1.01	0.91	0.91	1.14	1.01	**0.93b**	**0.99a**
	(14.6)	(15.8)	(60.5)	(75.9)	(19.9)	(19.2)	(5.3)	(5.4)	**(19.7)**	**(20.7)**
Mean	***0.95b***	***0.98a***	***0.88b***	***1.04a***	*0.90*	*0.92*	*1.15*	*1.06*	***0.92b***	***1.01a***
	(14.7)	***(15.9)***	***(61.9)***	***(78.1)***	*(19.6)*	*(19.3)*	*(5.3)*	*(5.6)*	***(19.5)***	***(21.1)***
	F_{10}	$F_{10.1}$	F_{10}	$F_{10.1}$	F_{10}	$F_{10.1}$	F_{10}	$F_{10.1}$	F_{10}	$F_{10.1}$
**G x S**										
Organic	**0.99b**	**1.04a**	1.07	0.97	**0.93b**	**1.00a**	**1.17a**	**1.20a**	**1.08b**	**1.06b**
	**(15.7)**	**(16.3)**	(77.0)	(71.1)	**(18.9)**	**(19.9)**	**(6.6)**	**(6.3)**	**(22.0)**	**(21.6)**
Conventional	**1.01b**	**0.99b**	1.07	1.04	**0.97a**	**0.98a**	**1.14a**	**1.04b**	**1.14a**	**1.05b**
	**(16.1)**	**(15.5)**	(77.3)	(76.0)	**(19.8)**	**(19.6)**	**(6.4)**	**(5.5)**	**(23.2)**	**(21.3)**
Mean	*1.00*	*1.02*	*1.07*	*1.01*	*0.95*	*0.99*	*1.16*	*1.12*	*1.11*	*1.06*
	(15.9)	*(15.9)*	*(77.2)*	*(73.6)*	*(19.4)*	*(19.8)*	*(6.5)*	*(5.9)*	*(22.6)*	*(21.5)*
**G x P**										
Y	**0.97c**	**0.97c**	1.08	0.98	**0.89c**	**0.95b**	**1.11b**	**1.01c**	**1.07b**	**0.99c**
	**(15.5)**	**(15.2)**	(77.9)	(71.2)	**(18.2)**	**(19.0)**	**(6.3)**	**(5.3)**	**(21.7)**	**(20.1)**
Q	**1.02b**	**1.07a**	1.06	1.03	**1.00a**	**1.01a**	**1.19ab**	**1.20a**	**1.14a**	**1.11ab**
	**(16.2)**	**(16.7)**	(75.9)	(76.0)	**(20.4)**	**(20.1)**	**(6.7)**	**(6.3)**	**(23.3)**	**(22.6)**
YQ	**1.00bc**	**1.02b**	1.08	1.00	**0.95b**	**1.01a**	**1.17ab**	**1.14ab**	**1.12a**	**1.07b**
	**(16.0)**	**(15.9)**	(77.6)	(73.5)	**(19.4)**	**(20.1)**	**(6.6)**	**(6.0)**	**(22.8)**	**(21.7)**
	F_{11}	$F_{11.1}$	F_{11}	$F_{11.1}$	F_{11}	$F_{11.1}$	F_{11}	$F_{11.1}$	F_{11}	$F_{11.1}$
**G x S**										
Organic	1.00	0.97	**0.89b**	**1.00a**	0.96	1.00	1.08	1.02	1.12	1.06
	(15.5)	(15.2)	**(64.9)**	**(75.2)**	(20.2)	(19.8)	(5.6)	(5.2)	(22.5)	(22.1)
Conventional	0.96	0.96	**0.87b**	**1.03a**	0.95	1.02	1.07	1.02	1.07	1.05
	(14.9)	(15.0)	**(63.1)**	**(77.4)**	(20.0)	(20.1)	(5.6)	(5.2)	(21.5)	(22.0)
	0.98	0.97	**0.88b**	**1.02a**	0.96	1.01	1.08	1.02	1.10	1.06
Mean	(15.2)	(15.1)	**(64.1)**	**(76.3)**	(20.1)	(20.0)	(5.6)	(5.2)	(22.0)	(22.1)

Means followed by different letters within a column of System and Population are significantly different ($P < 0.05$).
G x S: Generation x System; G x P: Generation x Populations;
Y: yield CCPs, Q: quality CCPs and YQ: yield by quality intercrossed CCPs
SRL - seminal root length; TRL – total root length; SL – shoot length; RDW– root dry weight; SDW –shoot dry weight
